# Radiation Exposure to Low-Dose Computed Tomography for Lung Cancer Screening: Should We Be Concerned?

**DOI:** 10.3390/tomography9010015

**Published:** 2023-01-24

**Authors:** Chiara Pozzessere, Christophe von Garnier, Catherine Beigelman-Aubry

**Affiliations:** 1 Department of Diagnostic and Interventional Radiology, Lausanne University Hospital (CHUV), 1011 Lausanne, Switzerland; 2 Faculty of Biology and Medicine, University of Lausanne (UNIL), 1011 Lausanne, Switzerland; 3 Division of Pulmonology, Department of Medicine, Lausanne University Hospital (CHUV), 1011 Lausanne, Switzerland

**Keywords:** computed tomography, diagnostic screening programs, early detection, lung, neoplasm, radiation dose-response relationship, radiation exposure

## Abstract

Lung cancer screening (LCS) programs through low-dose Computed Tomography (LDCT) are being implemented in several countries worldwide. Radiation exposure of healthy individuals due to prolonged CT screening rounds and, eventually, the additional examinations required in case of suspicious findings may represent a concern, thus eventually reducing the participation in an LCS program. Therefore, the present review aims to assess the potential radiation risk from LDCT in this setting, providing estimates of cumulative dose and radiation-related risk in LCS in order to improve awareness for an informed and complete attendance to the program. After summarizing the results of the international trials on LCS to introduce the benefits coming from the implementation of a dedicated program, the screening-related and participant-related factors determining the radiation risk will be introduced and their burden assessed. Finally, future directions for a personalized screening program as well as technical improvements to reduce the delivered dose will be presented.

## 1. Introduction

Lung cancer (LC) is the most common cancer in Europe and ranges second in the USA, accounting for nearly 2.2 million new cases per year and the leading cause of cancer death, with up to 1.8 million deaths in 2020 [1,2]. Despite substantial advances in our understanding of the pathogenesis, diagnostic techniques, and treatment options, overall LC prognosis still remains poor. The main reason is that diagnosis is frequently obtained at a locally advanced stage in up to 22% and at a metastatic stage in up to 57% of patients, at which the five-year survival rates are 32 and 6%, respectively [3]. Conversely, early diagnosis enables curative surgical treatment, increasing the five-year survival rate to 60% for localized disease that is, however, only diagnosed in 24% of patients [3]. Therefore, achieving an early diagnosis is a main objective for improving outcomes in the care of LC patients. 

The effectiveness of LC screening (LCS) has been explored by several randomized trials [4,5,6,7,8,9,10,11,12,13]. Although only the US National Lung Screening Trial (NLST) and the Nederlands-Leuvens Longkanker Screenings Onderzoek (NELSON) trial were adequately powered, cumulative evidence demonstrates reduction in LC-related mortality by screening smokers with low-dose computed tomography (LDCT) [4,5,6,7,8,9,10,11,12,13,14,15]. The NLST annually screened 53,454 smokers > 30 pack/years (p/y) and former smokers (<15 years) aged 55–75 over three rounds, reporting a 20% reduction of LC-related death compared with chest radiography as control arm [4]. In particular, in this trial, 52 and 11% of cancers were detected at stage IA and IB, respectively [4]. The observational NELSON trial screened 15,792 smokers >15 cigarettes a day >25 years or >10 cigarettes a day >30 years, as well as ≤10 years former smokers aged 50 to 74 over four screening rounds (at baseline, after 1, 2, and 2.5 years). This program allowed the detection of 187 cancers, 66% of which were at an early stage, with a reduction in LC-related mortality of 24% in males and 33% in females [7]. In particular, the cumulative LC detection was higher than the NLST (3.2 vs. 2.4%) [4,7]. Pooled analysis of the Multicentric Italian Lung Detection (MILD) and Detection and Screening of Early Lung Cancer by Novel Imaging Technology and Molecular Essays (DANTE) trials found a non-statistically significant 17% reduction in LC-related mortality [14]. A Cochrane systematic review of 11 US and European trials, including 91,122 participants, reported a 21% reduction in LC-related death, concluding that the impact of LDCT screening had moderate certainty of evidence [15]. Eligibility criteria, number of participants, type of study and the results of the main randomized clinical trials on LCS are reported in Table 1. Interestingly, a shift in the proportion of LC diagnosed at a localized stage was observed in the US following the introduction of LCS in the US Preventive Services Task Force (USPSTF) recommendations [16]. In particular, an increased incidence of 4.5% per year of early stages LC from 2013 was reported [3]. Consequently, professional societies and organizations worldwide are now committed to introducing LCS programs, potentially involving millions of individuals [17,18,19,20,21,22,23]. In addition, the implementation of a dedicated LDCT protocol within an LCS radiation dose range is expected for future concomitant assessment of the “Big-3”, i.e., lung nodule, emphysema and coronary artery calcium [17].

## 2. The Problem of Radiation Exposure in Lung Cancer Screening

Suitability of a screening program depends on the selection of a target risk population that would benefit from screening, the accuracy of the screening test, its frequency and duration, as well as the economic burden and adverse effects [24]. Radiation exposure is one of the major harms associated to LCS. Its potential oncological risk raises concerns and may dissuade eligible individuals to participate in an LCS program [25,26].

Several studies reported a lack of knowledge on radiation risk from medical imaging among both physician and general population, this frequently leading to a biased understanding of the benefit-to-risk balance of radiological examinations [27,28]. Therefore, raising awareness and improving information and communication on LCS radiation exposure is crucial for conscious adherence to the program [29].

While the secondary effects of high radiation doses are well known, those related to low-dose exposure by medical imaging are still debated. Based on epidemiological evidence from atomic bomb survivors, the National Academy of Sciences Biologic Effects of Ionizing Radiation (BEIR) VII report advanced a linear no-threshold (LNT) model to estimate the lifetime radiation-related risk of cancer [30]. The LNT model describes a linear and causal relationship between ionizing radiation and human cancer risk, lacking a threshold below which radiogenic cancer risk disappears [30]. To date, no association between low-dose (<100 mSv) irradiation and cancer has been demonstrated. While some expert working groups support the LNT model, others, based on experimental and epidemiological studies, hypothesize that the cancer risk from low-dose irradiation is extremely low [31]. Although the carcinogenic risk at low-dose still remains uncertain, attention must be paid to any radiation exposure, such as from medical imaging.

Hence, in LCS, ethical concerns do not only arise from exposing healthy individuals to radiation by cumulative radiation exposure from iterative prolonged screening rounds, but also from the additional radiological investigations required for the work-up of lesions detected by LDCT. Such further investigations may include follow-up LDCT, Positron Emission Tomography/Computed Tomography (PET/CT), fluoroscopy during bronchoscopic procedures, or CT-guided biopsy.

Several factors should be considered to estimate the risk related to radiation exposure in LCS, both screening-related (CT protocol, screening interval, duration) and participant-related (age at the start of the screening program, gender, tobacco exposure).

## 3. Radiation Dose Estimates in Lung Cancer Screening

### 3.1. Estimates of Single Low-Dose CT Round

As a rule, CT protocols are established to ensure diagnostic quality images while keeping a low radiation exposure according to the “as low as reasonably achievable” (ALARA) principle. For LCS, LDCT is recommended to reduce the effective dose (ED). ED (in milliSieverts, mSv) is a derived value that estimates dose exposure and the related biological risk. For CT examination, the ED is calculated by multiplying the dose-length product (DLP, in mGy x cm), which is the product of the volume computed tomography dose index (CTDIvol, in milliGrays, mGy) by the scan length (in centimeters, cm), using a specific conversion factor “k” of 0.0146 mSv/mGy x cm, according to IRCP103 [32].

In LCS trials, the ED ranges from 0.2 mSv to 2.36 mSv [4,10,33,34,35,36,37,38,39,40]. In the NLST, CTDIvol ranged from 3.02–3.81 mGy depending on participant’s body-weight, with an average ED estimate of 2 mSv per CT [4,41,42]. In the Nelson trial, the ED was <2 mSv, calculated for CTDIvol at 0.8, 1.6, and 3.2 mGy, depending on the subject’s body weight [7,43].

These single screening values that are lower than a regular chest CT (average of 3.8 mSv) are reassuring when considering individual average annual background dose of approximately 3–5 mSv [44,45]. Moreover, technical improvements in CT scanners over the past years have decreased the ED to approximately 0.7–1 mSv for routine examinations, such as follow-up for lung nodules. In conclusion, the estimated radiation-related risk of a single screening LDCT is presently substantially lower than ambient annual background irradiation.

### 3.2. Estimates of Cumulative Dose in Lung Cancer Screening

LCS over a prolonged period can enhance the screening benefit, leading up to a 58 and 39% reduction in LC mortality at 6 and 10 years, respectively [6]. Periodical LDCT leads to an increase in radiation-induced LC by an additional 1.8% [46]. Moreover, a positive LDCT may require further imaging by chest CT or PET/CT with an ED of approximately 3.8 mSv and 14 mSv, respectively [44,47,48]. Analyzing the NLST data, an average total radiation dose is estimated at 8 mSv per participant, with a lifetime radiation-induced risk of cancer based on the LNT model of 0.05 and 0.09% in male and female participants, respectively, leading to one radiation-induced cancer per 2500 subjects screened [47,49]. In the ITALUNG trial, the average ED was 1.2 mSv for a screening round, while the mean ED calculated on an average of 6 LDCTs over four years was 6.2–6.8 mSv [50]. However, in case of additional examinations, the maximal individual ED over four years may increase up to 19.5–21.5 mSv [50]. Rampinelli et al., analysing data from COSMOS study, calculated a median cumulative dose after 10 years of annual screening of 9 mSv and 13 mSv for men and women, respectively, corresponding to about a third of the natural background exposure [37]. The estimated lifetime attributable risk was 1.5 radiation-induced LC, corresponding to one radiation-induced LC for every 173 LC screen-detected (259 lung cancer detected/1.5 estimated lung cancer-induced) [37]. The estimates of the cumulative dose after a 25-year annual screening range were between 20.8 and 32.5 mSv [18].

### 3.3. Estimates Based on Interval Screening

Although cumulative dose may be reduced by biannual screening protocols, several societies recommend annual screening rounds [51,52,53,54]. The NELSON trial found no differences in LC detection in the third round for two-year interval screening compared to the annual interval [55]. The MILD trial also reported similar LC-related death when comparing annual and biannual screenings [56]. These results may be explained by the retrospective analysis of the MILD population, which showed that individuals with negative baseline LDCT had a 0.3% risk of LC two years afterward [57].

In contrast to the results from the initial screening rounds in the NELSON trial, the fourth round, performed 2.5 years after the third one, detected a significant number of interval LCs at an advanced stage, indicating that annual and biannual screenings are effective in early LC diagnosis, but increasing this to a 2.5-year interval may miss faster-growing lung cancers [55].

In conclusion, shifting to a biannual screening would save many LDCT rounds and reduce both cumulative dose exposure and costs. Consequently, future research should focus on personalized interval screening algorithms to identify participants that benefit from annual and biannual screening [57,58].

### 3.4. Estimates Based on Age at Start

The starting age differed among the trials, with 55-year-old in the NLST and ITALUNG trials versus 50-year-old in NELSON, DLCST, MILD and UKLS trials [4,6,7,8,10,11]. Currently, international societies recommend LCS in individuals between 55–79 years-old [16,22,51,52,53]. The USPSTF updated its recommendations in 2021, lowering the screening starting age by 5 years to 50 years to include more high-risk women and racial minorities, who are susceptible to developing LC at younger age [54].

The radiation-related risk depends on the age at start of screening. While for most tissues and organs radiation sensitivity is greatest at a younger age, lungs are more sensitive when exposed at an older age, with a peak around 50–55 years. The radiation-related LC risk hypothetically decreases from 1.8 to 0.8% by deferring the initiation of annual screening from 50 to 60 years [46]. The lifetime attributable risk of LC after 10 years of annual LDCT is 5.5 × 10^−4^ in a woman starting at 50 years compared to 5.1 × 10^−4^ at 55 years [37], and the benefit-risk balance is not affected at these ages, while it became distinctly outweighed by the radiation risk for ages <50 [59].

### 3.5. Estimates Based on Gender

There are actually gender discrepancies in both benefit and radiation risk in LDCT. Several studies demonstrated that LCS is more effective in women than in men since women more frequently develop slower growing adenocarcinomas [4,7,12,60,61]. In contrast, women are more sensitive to radiations and at risk of developing radiation-induced cancer than men, including a potential additional risk of radiation-induced breast cancer [37,44,46,62]. The lifetime attributable risk of cancer of a single LDCT for a standard-weighted 55-year-old individual is 0.6% and 0.3% in women and men, respectively [62]. This difference is more evident in prolonged LCS, with 0.1% estimated radiation-induced cancer in females compared to 0.05% in males when performing annual screening 55 to 80 years [62]. Thus, the balance between benefit and risk needs to be carefully weighed for women, especially when considering lowering the age for the initiation of LCS. In women, the lifetime attributable risk of major cancer after 10 years of annual LDCT changes from 8.1 × 10^−4^ at age 50 to 7.2 × 10^−4^ at age 55 [37]. The estimate of a simulating study calculated that entering prolonged biannual screening by 5 years earlier would allow for a 4% increase in LC screen-detection and at the cost of 55% increase in radiation-induced lung cancer, resulting in 1 radiation-induced cancer per 71 LCs detected [63]. Overall, these values are reassuring when assessing the benefit–risk ratio for the initiation of LCS to in 50-year-old participants.

### 3.6. The Synergic Role of Radiations and Tobacco

Smokers and former smokers are the target risk population for LCS. Carcinogenesis is increased in lungs exposed to tobacco and may synergistically augment the risk of developing cancer from radiation [46,64]. Dosimetry studies for LDCT measured an average dose delivered to the lungs of 1.5–4.5 mGy depending on the scanner technology, and up to 25 mGy during 10-years of annual screening [37,62,65]. These data substantiate the importance of tobacco cessation in LCS programs to minimize the synergistic carcinogenic risk from radiation and tobacco exposure.

## 4. Technical Approach of LDCT

Since the delivered ED depends on CT technology, facility protocol settings and patient’s body weight, guidelines setting the dose levels for LDCT in LCS have been published. In 2014, the American College of Radiology (ACR) recommended an upper limit for CTDIvol of 3 mGy for a standard-size patient with ≤1 mm thick images by using at least a 32 rows CT [51,66]. At these CTDIvol values, the ED in a standard-size patient would be about 1.0 mSv [26]. Currently, CT scanners with 32 rows and less are overcome by the new technologies and have been replaced by 64-row scanners or more. More recently, the European Society of Thoracic Imaging (ESTI) updated these recommendations following the technical improvements of hardware and software achieved in the last years [67]. Among the technical requirements, the ESTI indicates the use of at least a 64-row detector CT, and a CTDIvol of 0.4 mGy, 0.8 mGy, and 1.6 mGy for <50 kg, 50–80 kg, and >80 kg subjects, respectively, to select automatic exposure controls for kV selection and tube-current modulation depending on patient characteristics, and to reconstruct ≤1 mm thick slices, and employ iterative or deep learning reconstruction algorithms. These parameters would deliver an ED of approximately 0.7 mSv with diagnostic quality images [68].

The technical parameters recommended by the ESTI allow reduced radiation exposure when compared to those reported for most clinical trials; hence, the ED estimates for LCS should be lower than those reported to date.

However, the accomplishment of these dose values is not straightforward. A prospective analysis of data from 12,529 participants who underwent LCS in 72 institutions by Demb et al., observed that CTDIvol and ED exceeded the ACR benchmarks in up to 18 and 50% of the participants, respectively [69]. Higher doses were reported in older patients and women, depending on body weight. The authors also showed that the presence of an on-site dedicated medical physicist and a lead radiologist to establish the LDCT protocols were important factors for compliance with dose exposure guidelines. The optimization process of LDCT protocols should therefore integrate the expertise of an experienced chest radiologist and medical physicist. In addition, the radiologists and technologists involved in LCS should be trained in protocol acquisition to ensure optimal balancing between image quality and dose delivered for each examination.

## 5. Future Direction

### 5.1. Towards Individually Tailored Screening Programs

The main goal of LCS implementation is to identify the best strategy to obtain a cost-effective program.

In the USA, the implementation of LCS has encountered several issues, showing that a balance between invested effort and identification of individuals at risk, adherence to program and an acceptable benefit-harm-cost ratio is not as simple. Optimal participant selection, choice of screening intervals, and duration of program may reduce the potential issue and harms, such as participant dismissal, high costs, false positive results and excessive radiation exposure. The eligibility criteria for LCS in the USA rely on USPSTF2013 recommendations [16]. However, elaborate risk models, such as Prostate, Lung, Colorectal, and Ovarian model (PLCOm2012) are more efficient in identifying high-risk individuals than when using fixed criteria [70]. New risk prediction models provide an opportunity to establish tailored LCS and should be explored in future trials. In this setting, a multicentered trial conducted across five European countries, the “Towards INdividually tailored INvitations, screening INtervals and INtegrated co-morbidity reducing strategies in lung cancer screening” (4-IN-THE-LUNG-RUN), investigates a personalized risk-based approach, integrating tailored invitations to high-risk subjects based on risk factors, such as smoking status, sex and age, and individualized screening intervals taking into account the risk factors and CT results [71]. Such personalized approach is expected to have a positive impact on radiation exposure, as it will avoid unnecessary irradiation in lower-risk subjects [57,58].

### 5.2. Technological Advances

Recent developments in software and hardware technology have enabled low-noise images while maintaining diagnostic quality images, that allowing further dose reduction [68]. In particular, several studies have explored the acquisition of ultra-low dose CT (ULDCT) at sub-mSv dose levels and its impact on quality images and lung nodule assessment. When compared to LDCT, the diagnostic accuracy of ULDCT is slightly lower and, in particular, is dependent on nodule size and its intrinsic features. Sensitivity rates range between 61–99% and 59–100% for LDCT and ULDCT, respectively, while specificity is 100% and 81–100%, respectively [72]. In a study by Huber and colleagues, the ULDCT detection rate was lower than standard CT (93.3 vs. 95.5%, respectively) due to the under-detection of <5 mm solid and sub-solid nodules, which, however, was compensated by using maximum intensity projection (MIP) reconstructions and a computer-aided-detection (CAD) software [73]. Another study by Gheysens and colleagues evaluated nodule assessment of ULDCT by using a scoutless CT at a fixed CTDIvol of 0.15 mGy [74]. The authors found that nodule detection rate was comparable between standard CT and ULDCT: 76 and 78%, respectively; however detection significantly relied on nodule size, since up to 20% of <5 mm nodules were missed by ULDCT [74]. Due to the blurred edges, the nodule measurements were 9% lower compared to standard CT; given that this value is below the interscan variability, this difference would not affect the nodule management [74]. However, up to 10% of solid nodules were misdiagnosed as sub-solid nodules at ULDCT, potentially compromising their further management [74].

Interesting results come by deep learning iterative reconstruction (DLIR), an artificial intelligence method for CT image reconstruction that reduces background image noise, allowing image quality improvement and, consequently, a dose reduction of about 20% [75]. In a prospective study on ULDCT, the nodule detection rate was better when using the DLIR images compared to adaptive statistical iterative reconstruction (ASIR), the most used reconstruction algorithm: 75.8 vs. 73.3%, respectively [75]. Nevertheless, further technological improvements are still required before these ultra-low doses can be applied to clinical activity. It could be hypothesized that, in the future, ULDCT would find a place in LCS and could be alternatively performed in screening rounds in some conditions to reduce radiation exposure of long-term screening protocols.

A new CT technology using photon-counting detectors (PCD) is under evaluation [76]. PDCT-CT releases electric pulse signals that are converted from X-rays, increasing CT performance by decreasing image noise and radiation dose [77]. Preclinical studies showed promising results; in particular, the higher resolution of PDCT allowed for better morphological and volumetric nodule assessments when compared to conventional CT [78].

Finally, the value of magnetic resonance imaging (MRI) in nodule detection and volume assessment should also be explored, especially with low-field equipment at 0.55 T [79,80]. A pilot study by Delacoste et al., observed similar performances between CT and Ultra-short echo (UTE) MRI when quantifying lung nodule volumes using both human and artificial models [81].

## 6. Conclusions

In conclusion, currently available data from LCS trials and simulation studies are reassuring in terms of radiation burden from LDCT. Nevertheless, attention must be paid to the accurate selection of eligible subjects who are at the highest risk and the optimal CT protocols to minimize radiation-induced cancer risk during LCS rounds and additional investigations. Personalized screening algorithms and risk stratification, taking into account gender, age, interval time and duration are desirable to reduce unnecessary radiation exposure. A dedicated radiologist and medical physicist should optimize the LDCT protocol to comply with international guidelines and ensure an optimal image quality/radiation dose balance. Future technological advances that will improve image quality will probably allow further reduction on delivered dose in this setting.

## Figures and Tables

**Table 1:** Eligibility criteria, number of participants, type of study, and results of the main randomized clinical trials on lung cancer screening. NLST and NELSON trials are the only powered studies. Pooled results of MILD and DANTE show 17% LC mortality reduction (HR 0.8).

Trial	Age (y/o)	Smoking Status	*n*	Type of Study	Rounds and Follow-Up	Results
NLST	55–75	smokers >30 p/y; <15 years former smokers	53,454	LDCT vs. CXR	3 annual rounds; 5.2 years	20% reduced LC mortality compared to CXR (HR 0.8, *p* < 0.004)
NELSON	55–74	>15 p/y; ≤10 years former smokers	15,792	LDCT observational	4 rounds (1, 2, and 2.5 years after); 11 years	24% and 33% reduced LC mortality in males and females, respectively (HR 0.76)
DANTE	60–74	>20 p/y; <10 years former smoker	2811	LDCT observational	4 annual rounds; 8 years	Non-significant reduction in LC mortality (HR 0.99)
MILD	>49	>20 p/y; <15 years former smoker	4099	LDCT observational	8 years annual or biannual rounds; 10 years	Reduction of 10-year risk of LC mortality (HR 0.61)
ITALUNG	55–69	>20 p/y; <10 years former smoker	3206	LDCT observational	4 annual rounds; 10 years	Non-significant reduction in LC mortality (HR 0.7)
DLCST	50–70	>20 p/y; <10 years former smoker	4104	LDCT vs. CXR	5 annual rounds; 5 years	Non-significant reduction in LC mortality (HR 1.03)
LUSI	50–69	>15 p/y; <10 years former smoker	4052	LDCT observational	5 annual rounds; 8.8 years	Reduction in LC mortality in women (HR 0.31; *p* = 0.04)
UKLS	50–75	N/A	4055	LDCT observational	1 round; 7.3 years	Non-significant reduction in LC mortality (HR 0.65)

NLST, National Lung Screening Trial; NELSON, Nederlands-Leuvens Longkanker Screenings Onderzoek; DANTE, Detection and Screening of Early Lung Cancer by Novel Imaging Technology and Molecular Essays; MILD, Multicentric Italian Lung Detection; DLCST.

## Abbreviations

4-IN-THE-LUNG-RUN	Towards INdividually tailored INvitations, screening INtervals and INtegrated co-morbidity reducing strategies in lung cancer screening
ACR	American College of Radiology LC: Lung Cancer
ALARA	As Low as Reasonably Achievable
ASIR	Adaptive Statistical Iterative Reconstruction
BEIR	Biologic Effects of Ionizing Radiation
CAD	Computer-aided detection
CTDIvol	Volume Computed Tomography Dose Index
DANTE	Detection and Screening of Early Lung Cancer by Novel Imaging Technology and Molecular Essays
DLIR	Deep Learning Iterative Reconstruction
ED	Effective dose
ESTI	European Society of Thoracic Imaging
LCS	Lung Cancer Screening
LDCT	Low-Dose Computed Tomography
LNT	Linear No-Threshold
MILD	Multicentric Italian Lung Detection
MIP	Maximum intensity projection
NELSON	Nederlands–Leuvens Longkanker Screenings Onderzoek
NLST	National Lung Screening Trial
P/Y	Pack/Years
PCDCT	Photon-Counting Detectors Computer Tomography
PET/CT	Positron Emission Tomography/Computed Tomography
PLCOm2012	Prostate, Lung, Colorectal, and Ovarian model
USPSTF	United States Preventive Services Task Force
